# Do We Need Exercise Tests to Detect Gas Exchange Impairment in Fibrotic Idiopathic Interstitial Pneumonias?

**DOI:** 10.1155/2012/657180

**Published:** 2012-07-29

**Authors:** Benoit Wallaert, Lidwine Wemeau-Stervinou, Julia Salleron, Isabelle Tillie-Leblond, Thierry Perez

**Affiliations:** ^1^Clinique des Maladies Respiratoires, Centre de Compétence des Maladies Pulmonaires Rares, Hopital Calmette, Lille 2 University Boulevard Leclercq, CHRU, 59037 Lille, France; ^2^Unité de Biostatistiques, CHRU, 59037 Lille, France; ^3^Service d'Explorations Fonctionnelles Respiratoires, Hopital Calmette, CHRU, 59037 Lille, France

## Abstract

In patients with fibrotic idiopathic interstitial pneumonia (f-IIP), the diffusing capacity for carbon monoxide (DLCO) has been used to predict abnormal gas exchange in the lung. However, abnormal values for arterial blood gases during exercise are likely to be the most sensitive manifestations of lung disease. The aim of this study was to compare DLCO, resting PaO_2_, P(A-a)O_2_ at cardiopulmonary exercise testing peak, and oxygen desaturation during a 6-min walk test (6MWT). Results were obtained in 121 patients with idiopathic pulmonary fibrosis (IPF, *n* = 88) and fibrotic nonspecific interstitial pneumonias (NSIP, *n* = 33). All but 3 patients (97.5%) had low DLCO values (<LLN) whereas only 66.6% had low KCO; 42 patients (65%) exhibited resting hypoxemia (<75 mmHg); 112 patients (92.5%) exhibited a high P[(A-a)O_2_], peak (>35 mmHg) and 100 (83%) demonstrated significant oxygen desaturation during 6MWT (>4%). Interestingly 27 patients had low DLCO and normal P(A-a)O_2_, peak and/or no desaturation during the 6MWT. The 3 patients with normal DLCO also had normal PaO_2_, normal P(A-a)O_2_, peak, and normal oxygen saturation during 6MWT. Our results demonstrate that in fibrotic IIP, DLCO better defines impairment of pulmonary gas exchange than resting PaO_2_, exercise P(A-a)O_2_, peak, or 6MWT SpO_2_.

## 1. Introduction

According to the ATS/ERS statement, fibrotic interstitial idiopathic pneumonia (f-IIP) includes idiopathic pulmonary fibrosis (IPF) and fibrotic nonspecific interstitial pneumonia (f-NSIP) [1–4]. Although pathological abnormalities are quite different between these two diseases [5], alteration of gas exchange is a major abnormality which is thought to reflect the severity of fibrotic process [6].

Given the simplicity of pulmonary function testing, many investigators have examined the potential for simple resting physiologic measurements to stratify disease severity. The classic physiologic findings in the fibrotic IIP include a reduction in lung volumes (vital capacity; total lung capacity), a reduction in carbon monoxide diffusing capacity (DLCO), and hypoxemia that worsens with exercise [2]. Evaluation of gas exchange impairment can be performed in clinical practice by simple tests like DLCO, resting PaO_2_, and P(A-a)O_2_, measurement of SpO_2_ during a 6-min walk test (6MWT) or PaO_2_ and alveolar-arterial oxygen pressure difference P(A-a)O_2_ during cardiopulmonary exercise testing (CPET).

Whereas DLCO is a valuable tool in the assessment of the efficiency of pulmonary gas exchange, the P(A-a)O_2_, especially during exercise, is thought to better reflect the normality of respiratory gas exchange [8, 9]. In addition exercise-induced gas exchange can also be readily identified by simple testing such as the 6MWT [10].

To the best of our knowledge, comparison of all the various methods to detect pulmonary gas exchange abnormalities has never been performed. Previous studies compared DLCO and P(A-a)O_2_ in a small number of IPF patients but did not include 6MWT [9, 11] or analysed 6MWT oxygen desaturation but did not include analysis of exercise PaO_2_ or P(A-a)O_2_ [12, 13]. With this in mind we performed a retrospective analysis of resting and exercise tests in 138 consecutive patients with IPF or f-NSIP.

## 2. Patients and Methods

One hundred and thirty eight caucasian patients with a diagnosis of IPF or f-NSIP were consecutively referred for evaluation of dyspnea and CPET at the time of diagnosis or during followup, over a period of six years. Inclusion criteria consisted of diagnosis of IPF according to the American Thoracic Society/European Respiratory Society guidelines and/or histopathological evidence for usual interstitial pneumonia, or diagnosis of f-NSIP (radiographic or histopathological diagnosis) [1, 2]. Patients were not included if they had another pulmonary disease (including obstructive disease), left heart failure or a history of pulmonary embolism. Connective tissue diseases were ruled out. No acute exacerbation was observed in the three months preceding inclusion. Seventeen patients were excluded from the study because CPET was not performed (arthrosis). Therefore 121 patients (31 females, 90 males) were included. In 44 out of the 88 IPF patients and 20 out of the 33 patients with f-NSIP, diagnosis was confirmed by histopathological examination of lung biopsy. At the time of inclusion in the study, a majority of patients (76%) were not treated, 19 patients received corticosteroids, 12 patient received azathioprine, and 3 patients received mycophenolate mofetil. Clinical data and results of pulmonary function tests, 6MWT, and of CPET were collected. Only initial data were recorded when the patient was seen several times. Approval for the use of these data was provided by the Institutional Review Board of the French learned society for respiratory medicine (CEPRO 2011-039).

## 3. Pulmonary Function Tests

Forced vital capacity (FVC), forced expiratory volume in 1 second (FEV_1_), and total lung capacity (TLC) were measured by spirometry and plethysmography with a Jaeger-Master lab cabin. Single-breath diffusing capacity of the lung for carbon monoxide (DLCO: mLCO·min^−1^·mmHg^−1^) and carbon monoxide transfer coefficient (KCO = DLCO/alveolar volume) were measured. DLCO was corrected for hemoglobin concentration in g·dL^−1^, according to Cotes' equation: corrected (Hb) DLCO = DLCO × (10.2 + Hb)/(1.7 × Hb). Values were expressed as percentages of the predicted normal values calculated according to gender, weight, and age. Reference equations for spirometry were taken from ERS for lung volumes and DLCO [14, 15]. Following ATS/ERS 2005 guidelines, the lower limits of normal (LLN) were set at the level of 5th percentile (or predicted minus 1.64 SD) of each reference population [16]. Results were conventionally expressed as percent predicted.

The 6MWT was performed in accordance with international recommendations [17] and was designed to ensure an accurate assessment of oxygen desaturation Patients were instructed as follows: “The object of this test is to walk as quickly as you can for 6 minutes to cover as much ground as possible. You may slow down if necessary. If you stop we wish you to continue the walk again as soon as possible. Your goal is to walk as fast and as far as you can in 6 minutes.” [18]. The pulse oximeter was lightweight, battery powered, and held in place by a “fanny pack” so that the patient does not have to hold or stabilize it. We evaluated the oxygen saturation at rest and the lowest saturation during the test. A desaturation ≥4% was considered as significant [2].

CPET was carried out using a standardized protocol as previously described [19] and consisted of a triangular test, carried out on an ergometric bicycle (Ergoline-Ergometrics 800). Briefly the expired gases were determined in each cycle with an Ergocard. During exercise, heart rate (HR) was monitored continually by 12-lead ECG and arterial oxygen saturation (SpO_2_) was measured by pulse oximetry with a Nellcor N-395 apparatus. Arterial blood samples were obtained from a small catheter placed in the radial artery under local anesthesia. Measurements of PaO_2_ and PaCO_2_ were performed on room air at rest and at peak exercise. Normal values for PaO_2_ were derived from Sorbini et al. [20]. The alveolar-arterial gradient in oxygen [P(A-a)O_2_] was calculated from the alveolar gas equation. According to ATS statement, [P(A-a)O_2_], peak >35 mmHg was considered as abnormal [21]. Exercise pulmonary gas exchange variables were either related or not related to the metabolic demand (VO_2_), that is, peak exercise-rest (Δ) [19, 22]. The modified Bohr equation was used to calculate dead space to tidal volume ratio (VD/VT). Predicted values were calculated from reference equations [22, 23]. Poor motivation was not a factor interfering with our analysis as suggested by the fact that all of the patients had one or more of the following criteria: breathing reserve less than 15%, peak HR more than 90% of predicted, peak lactate more than 7 mEq/L, peak exercise PaO_2_ less than 55 mmHg or peak VE/VO_2_ more than 35or RER >1.15 [24, 25].

## 4. Statistical Analysis

After certification of normal distribution, data are reported as mean ± SD. Student's *t*-test was used to determine differences between IPF and f-NSIP. Differences in proportions were assessed by *χ*2 tests. Correlations were analysed using Spearman's rank correlation test. All statistical analysises were carried out with GraphPad Prism 4.0 software (San Diego, Calif, USA). Values of *P* < 0.05 were considered significant.

## 5. Results

Characteristics of the population are summarized in Table 1. The overall population consisted of 90 men and 31 women with a mean age of 63.6 ± 8.4 years: 88 patients had a diagnostic of IPF and 33 of f-NSIP. Pulmonary function tests results are shown in Tables 1 and 2. As expected, we observed a reduction in TLC, VC, and FEV_1_, a reduced DLCO and KCO. DLCO was reduced to a greater extent than the lung volumes: 45 out of 121 patients (37%) showed a normal FVC and 24% a normal TLC despite a low DLCO.

All but 3 Patients (97.5%) had low DLCO values (<LLN, corresponding to a mean 73 ± 0.4% predicted) whereas only 66.6% had a low KCO; 42% patients exhibited resting hypoxemia (<75 mmHg) and 26% a high resting P(A-a)O_2_; 112 patients (92.5%) exhibited an increased P[(A-a)O_2_], peak, 83% a high ΔP(A-a)O_2_/ΔVO_2_ and 100 patients (83%) demonstrated significant O_2_ desaturation during 6MWT. There was no significant difference between IPF and f-NSIP for all parameters except VD/VT peak which was higher in IPF (*P* = 0.01).

DLCO was severely reduced in the 79 patients with normal resting PaO_2_. Interestingly 27 patients had low DLCO and normal P(A-a)O_2_, peak and/or no desaturation during the 6MWT. Nine patients had normal P(A-a)O_2_, peak: 6 out of 9 did not show significant desaturation during walk test. Conversely among the 21 patients with low DLCO and without significant desaturation at the 6MWT, all had abnormal P(A-a)O_2_, peak. The 3 patients with normal DLCO also had normal PaO_2_, normal P(A-a)O_2_, peak, and normal oxygen saturation during 6MWT.

**Figure 1:**
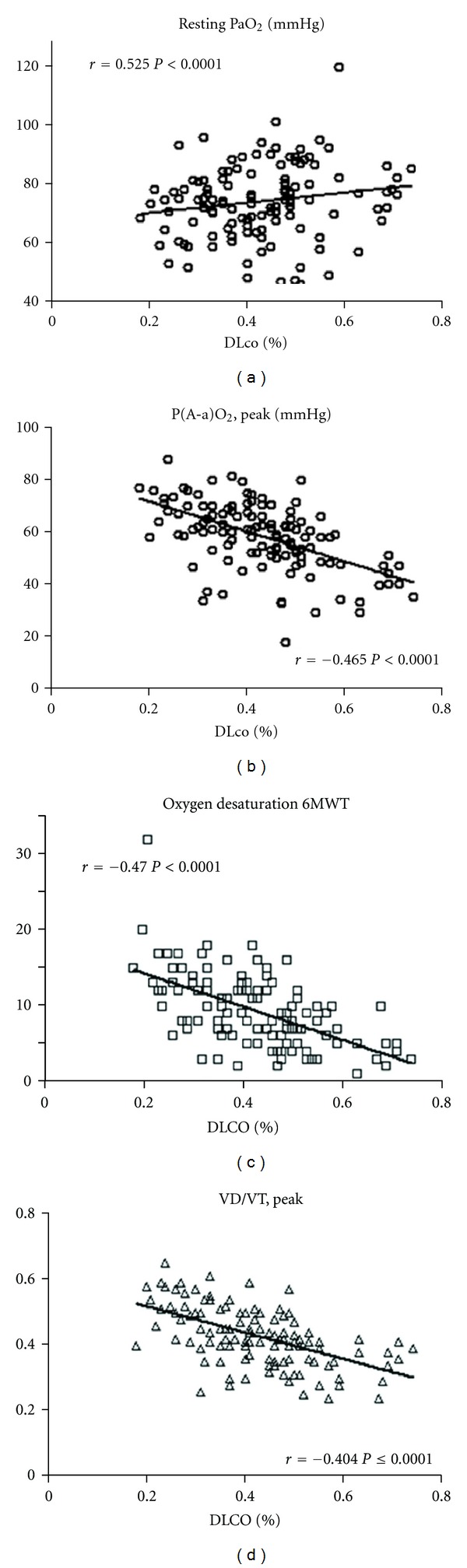
Correlation between DLCO (percent predicted) and resting PaO_2_, P(A-a)O_2_, peak, oxygen desaturation during 6MWT and VD/VT, peak in fibrotic IIP.

We found a very good correlation between DLCO and lung volumes and other measures of gas exchange (Table 3 and Figure 1). Interestingly we also found a good correlation between DLCO and VD/VT.

Resting parameters and indexes of gas exchange were more severely altered according to disease severity as judged on alteration of DLCO (Table 4).

## 6. Discussion

There were three main findings in this study: first, abnormal gas exchange is present in patients with normal lung volumes; second, a low DLCO was found in 97.5% patients with f-IIP whereas resting PaO_2_, 6MWT oxygen desaturation and P(A-a)O_2_, peak were abnormal, respectively, in 42%, 83%, and 92.5%; and third, no patient had normal DLCO and abnormal PaO_2_, 6MWT oxygen desaturation, or increased P(A-a)O_2_, peak. As a consequence, DLCO is more sensitive for demonstrating gas exchange abnormality in fibrotic IIP than resting PaO_2_, exercise P(A-a)O_2_, peak, or 6MWT SpO_2_.

Clearly, DLCO is reduced in a greater extent than lung volumes in f-IIP and therefore abnormal DLCO is a frequent finding in patients with normal lung volumes. This has been demonstrated in previous studies [26, 27], both in IPF and f-NSIP [28–39]. Along this line, Gaensler and coworkers noted a fair correlation between histologic severity and physiologic indices [38]. Crystal and colleagues reported a poor correlation with spirometry, lung volumes, DLCO, and resting gas exchange in IPF [39]. In 14 untreated patients with IPF, DLCO, and lung volumes correlated with the extent of fibrosis and cellular infiltration; both of these correlated more strongly than gas exchange with exercise [6].

In patients with f-IIP, the DLCO has been widely used to predict abnormal gas exchange in the lung. Resting PaO_2_ correlates poorly with disease severity. In our studies, resting PaO_2_ was in the normal range in 58% cases. In contrast, abnormal values for arterial blood gases during exercise are more sensitive than resting PaO_2_. However our study in a large group of patients demonstrated that patients with abnormal gas exchange during exercise always exhibited abnormal DLCO and that, in contrast, abnormal DLCO was found in patients with normal gas exchange during exercise. A significant 6MWT oxygen desaturation and/or an increased P(A-a)O_2_ was never observed in f-IIP patients with normal DLCO whereas this has been previously reported in sarcoidosis [40, 41].

 Our 6MWT results are in agreement with the results of Lama and coworkers [18] who reported 6MWT oxygen desaturation results in IPF and NSIP: in this study, 80% IPF patients and 64% NSIP patients exhibited an oxygen desaturation ≥4% during 6MWT. The 6MWT is a noninvasive, cheap, and simple field test to carry out and interpret. However despite these advantages, some variabilities in the results obtained are observed [42] and an increased ventilatory response during 6MWT might be responsible for higher PAO_2_ minimizing the decrease in SaO_2_.

Factors that contribute to reduction in DLCO include abnormal thickness of the alveolar capillary membrane and reduced pulmonary capillary blood volume. Thus, DLCO is highly dependent on pulmonary vascular blood volume. We recently reported in patients with f-IIP that the Vc component of the DLCO was significantly decreased in addition to the already lowered Dm, CO component as a consequence of the thickened membranes [43]. The correlation between DLCO and VD/VT, peak is in agreement with the findings by Agusti and coworkers [8] and supports the concept that the abnormalities of the pulmonary vasculature are important to modulate gas exchange in IPF during exercise.

It was not the scope of our study to evaluate the prognostic value of each test. Several studies found that distance or desaturation during a 6MWT was a strong predictor of mortality [18]. Mortality rate is higher among patients with DLCO <30% to 45% predicted [33, 44–46], but it is clear that the prognostic value of any pulmonary functional parameter at one point is limited.

In conclusion, DLCO appears as the best physiologic index to evaluate gas exchange abnormalities in fibrotic IIP and could take the place of formal exercise testing with arterial blood gas to evaluate the severity of gas exchange in patients with fibrotic IIP.

## Figures and Tables

**Table 1:** Pulmonary function tests results.

	All patients	IPF	f-NSIP
	*n* = 121	*n* = 88	*n* = 33
Age	63.6 ± 8.4	64.3 ± 8.3	61.6 ± 8.5
BMI	28.4 ± 4.4	28.2 ± 4.1	29.1 ± 5.1
TLC (L)	4.35 ± 1	4.39 ± 1.01	4.23 ± 1
TLC (%)	70 ± 14.5	69.4 ± 14.6	73 ± 13.9
% with low TLC	76	82	62
FVC (L)	2.72 ± 0.74	2.77 ± 0.72	2.61 ± 0.8
FVC (%)	76 ± 16	75.5 ± 16.9	77.2 ± 13.6
% with low FVC	61	58	65
FEV_1_ (L/sec)	2.24 ± 0.58	2.26 ± 0.57	2.12 ± 0.6
FEV_1_ (%)	78.5 ± 16.4	78.4 ± 17.2	78.8 ± 14.1
% with low FEV_1_	55	52	62
DLCO (mLCO·min^−1^·mmHg^−1^)	11.2 ± 4	11.17 ± 4.4	11.3 ± 3.2
DLCO (%)	42.9 ± 12.3	41.9 ± 12.5	45.6 ± 11.6
% with low DLCO	98	98	97
KCO (mLCO·min^−1^·mmHg^−1^/L)	2.99 ± 0.76	2.92 ± 0.77	3.2 ± 0.7
KCO (%)	71.3 ± 17	70.3 ± 17.7	74.2 ± 3.7
% with low KCO	66.6	70	56
PaO_2_, rest (mmHg)	76.8 ± 12.6	75.9 ± 12.8	79.4 ± 12
% with low PaO_2_	42	46	31
P(A-a) O_2_, rest (mmHg)	30.8 ± 12.5	31.4 ± 12.6	29.2 ± 12.2
% with low P(A-a) O_2_, rest	26	30	16

**Table 2:** Cardiopulmonary exercise testing and walking test results.

	All patients	IPF	f-NSIP
	*n* = 121	*n* = 88	*n* = 33
Workload, peak (Watts)	81.4 ± 24	82.5 ± 23.7	78.3 ± 25.9
Workload, peak (%)	71.3 ± 9.9	61.2 ± 18.8	67.2 ± 24.8
VO_2_, peak (mL/Kg/min)	15.9 ± 3.9	15.9 ± 3.6	15.9 ± 4.6
VO_2_, peak (%)	66.5 ± 15.7	66 ± 15	67.8 ± 17.7
% low VO2, peak (%)	84	87	80
PaO_2_, peak (mmHg)	57.9 ± 13	56.6 ± 12.9	61.6 ± 12.6
ΔPaO_2_ (mmHg)	18.9 ± 8.3	19.4 ± 8.5	17.6 ± 7.8
P(A-a)O_2_, peak (mmHg)	58.1 ± 13	58.9 ± 13.2	61.7 ± 12.7
% with high P(A-a)O_2_, peak	92.5	95	84
ΔP(A-a)O_2_/ΔVO_2_ (mmHg/L)	34.2 ± 16.9	34.4 ± 17.1	33.6 ± 16.7
% with high ΔP(A-a)O_2_/ΔVO_2_	83	82	84
VD/VT, peak	0.43 ± 0.09	0.44 ± 0.09	0.39 ± 0.08^∗^
Walk test, distance (m)	388 ± 102	393 ± 98	375 ± 114
Walt test, nadir SaO_2_ (%)	86 ± 5.7	85.6 ± 6	88.3 ± 4.6
Walk test, ΔSaO_2_ (%)	9.2 ± 4.7	9.7 ± 5	7.9 ± 3.7
% with ΔSaO_2_ ≥4%	83	83	84

^∗^Significantly different from IPF group (*P* = 0.01).

**Table 3:** Correlation between percent-predicted DLco and other measures.

Variable	coefficient	*P* value
FVC	0.56	<0.0001
TLC	0.437	<0.0001
FEV_1_	0.508	<0.0001
Kco	0.56	<0.0001
Resting PaO_2_	0.525	<0.0001
P(A-a)O_2_, peak (mmHg)	−0.465	<0.0001
ΔP(A-a)O_2_/ ΔVO_2_ (mmHg/L)	−0.534	<0.0001
6MWT, nadir SpO_2_ (%)	0.511	<0.0001
6MWT, ΔSpO_2_ (%)	−0.47	<0.0001
VD/VT, peak	−0.404	<0.0001

**Table 4:** Rest and exercise parameters as a function of disease severity defined by DLco%: mild: DLco ≥ 60%, moderate: DLco < 60% and ≥40% and severe (advanced disease): DLco < 40% [7].

Disease severity	Mild	Moderate	Severe
Parameter	Dlco ≥ 60%	40% ≤ DLco < 60%	DLco < 40%
	*n* = 10	*n* = 65	*n* = 46
FVC	99 ± 20	77 ± 11^∗^	69 ± 16^$∗∗^
FEV1	99 ± 19	81 ± 12^∗^	70 ± 16^$*£*^
TLC	89 ± 13	70 ± 11^∗^	66 ± 16^$^
PaO_2_, rest (mmHg)	89 ± 6.4	80 ± 9^∗^	69 ± 13.7^$∗∗^
PaO_2_, peak (mmHg)	78.7 ± 9	59 ± 10^∗^	51 ± 11^$∗∗^
P(A-a)O_2_, rest (mmHg)	22 ± 9	27 ± 9.8	38 ± 13^$∗∗^
P(A-a)O_2_, peak (mmHg)	40 ± 7	57 ± 12^∗^	64 ± 12^$*£*^
ΔP(A-a)O_2_/ΔVO_2_ (mmHg/L)	14.8 ± 11	31 ± 12.5^∗^	44 ± 18^$∗∗^
Walt test, nadir SaO_2_ (%)	92 ± 3	87 ± 4^∗^	83 ± 6^$∗∗^
Walk test, ΔSaO_2_ (%)	4.3 ± 2.4	8.4 ± 3.8^∗^	11.5 ± 5.2^$∗∗^

Significantly different from group moderate ^*£*^ *P* < 0.001, ^∗∗^ *P* < 0.01.

Significantly different from group mild ^$^ *P* < 0.001, ^∗^ *P* < 0.01.
